# Evaluation of dental plaque reduction using microcurrent-emitting toothbrushes in orthodontic patients: a randomized, double-blind, crossover clinical trial

**DOI:** 10.1038/s41598-024-60753-9

**Published:** 2024-05-27

**Authors:** Ji-Hoi Kim, Jae-Hun Yu, Utkarsh Mangal, Jing Liu, Hyo-Jung Jung, Jung-Yul Cha

**Affiliations:** 1 Department of Orthodontics, Institute of Craniofacial Deformity, Yonsei University College of Dentistry, Seoul, Korea; 2 BK21 FOUR Project, Yonsei University College of Dentistry, Seoul, Korea; 3 Department of Orofacial Pain and Oral Medicine, Dental Hospital, Yonsei University College of Dentistry, Seoul, Korea; 4 Department of Orthodontics, Institute of Craniofacial Deformity, College of Dentistry, Institute for Innovation in Digital Healthcare, Yonsei University, Seoul, Korea

**Keywords:** Health care, Medical research

## Abstract

This study aimed to compare the effectiveness of microcurrent-emitting toothbrushes (MCTs) and ordinary toothbrushes in reducing the dental plaque index (PI) and dental caries activity among orthodontic patients. The evaluation was performed using a crossover study design involving 22 orthodontic patients randomly assigned to the MCT or ordinary toothbrush groups. The participants used the designated toothbrush for 4 weeks and had a 1-week wash-out time before crossover to the other toothbrush. PI (Attin’s index) and dental caries activity were measured at baseline and at the end of each 4-week period. Additionally, patients completed questionnaires to assess patient satisfaction for “freshness in mouth” and “cleansing degree.” The results showed that the MCT group had a significant reduction in PI (p = 0.009), whereas the ordinary toothbrush group did not (p = 0.595). There was no significant difference in the dental caries activity between the two groups (p > 0.05). Patient satisfaction assessment revealed that 65% patients in the MCT group had more than “fair” experience of freshness, in contrast to 50% of patients in the ordinary toothbrush group. Satisfaction with cleansing degree was similar in both groups. Overall, these findings suggest that MCTs are more effective in reducing dental PI than ordinary toothbrushes.

## Introduction

The accumulation of dental plaque is a key etiological factor in enamel demineralization and periapical gingivitis. This is particularly aggravated in orthodontic patients, for whom efficient plaque removal is difficult due to fixed appliances. The site of bracket placement and the immediate tooth interface are major sites for the accumulation of dental plaque, even after brushing, increasing the occurrence of enamel demineralization^1^. Dental plaque around the orthodontic appliance system can also result in an increase in the total microbial population with changes in the microbial ecosystem, leading to chronic infections, including periodontitis^2–4^.

Several methods have been proposed for managing plaque-induced diseases in patients receiving long-term orthodontic care. The use of special bristles with short toothbrush heads and adjunctive oral hygiene therapy, such as chlorhexidine mouthwash, fluoride varnish application, and the use of super-floss, are actively practiced clinically^5^. The mechanical method with a short-bristle brush and the use of Charter’s technique (rotating the toothbrush face tilted 45° back and forth) are believed to improve access around the oral cavity, whereas the adjunct methods are aimed at a chemical effect. Nonetheless, most oral hygiene practices are compliance-dependent, and an instant change in brushing habits or the use of interdental cleaning aids may result in a lower adaptation. Furthermore, prescriptions for hygiene management during orthodontic treatment vary by age group; therefore, there is no universally applicable hygiene method. In other words, an effective alternative that is easier to adapt to address plaque-induced problems is necessary.

Numerous studies have reported short- and long-term comparisons of the efficacies of powered toothbrushes. Electric toothbrushes are more effective than manual toothbrushes in removing plaque and controlling gingivitis^6^. An in vitro study also reported that an electric toothbrush significantly penetrated the interproximal area beyond the reach of the bristles^7^. Although in vitro biofilm removal with the use of electric toothbrushes is promising, other risks have been reported, such as gingival recession and tooth abrasion due to electrical vibration^8,9^. In addition, a clear conclusion could not be drawn as to whether electric or manual toothbrushes were most effective for biofilm management in orthodontic patients^10^. Similarly, no change in plaque index (PI) or gingival index (GI) with an electric toothbrush was reported after 4 and 8 weeks of use in orthodontic patients^11^.

To effectively disrupt biofilms and mitigate the pathogenic environment within the oral cavity, it is imperative to remove the extracellular polymeric substance (EPS) matrix^12,13^.

Recently, a new biofilm management device that applies the bioelectric (BE) concept was proposed. The BE concept can be applied using a toothbrush with an electric circuit to deliver alternating and direct microcurrents from the bristles. Microcurrents generate 10 million microwaves per second and are safe for humans. The microcurrent is the magnetic field formed at a toothbrush head radius of 2 cm. This is safe, with 100-µA current that is weaker than the biological current of 1000 µA^14^. Such stimulation is believed to cause biochemical inhibition of microbial metabolism and electrostatic detachment of biofilms from the tooth surface^15^. According to in vitro analyses, microcurrents reduce bacterial biomass by 88.15%, indicating a significant effect in removing biofilm^16,17^. A previous study analyzed fluorapatite generation in enamel using microcurrent-emitting toothbrushes (MCTs), and the results showed that MCTs enhanced the fluoride content on the enamel surface by 22.29% (spectroscopy analysis) compared to the small percentage (1.76%) with the use of an ordinary brush^18^. Another study on the effects of MCTs on plaque removal showed a 1.75 times higher reduction in the GI among 40 clinical participants^14^. However, the majority of the data are presented for general applications, and their effectiveness in otherwise complex conditions, such as with fixed orthodontic appliances, has not been investigated.

Therefore, the aim of the present study was to evaluate the efficacy of MCTs in removing dental plaque in patients undergoing fixed orthodontic treatment. We evaluated the PI and caries activity to confirm the effect of MCTs on oral health during orthodontic treatment. Furthermore, we assessed the level of satisfaction among orthodontic patients regarding the maintenance of personal oral hygiene while using an MCT.

## Methods

### Study participants and sample size calculation

^19^.

A double-blind, randomized, crossover study was conducted on patients receiving orthodontic treatment after visiting the Yonsei University Dental College Hospital in January 2019. The effect size (0.7276878) was calculated based on the average value and standard deviation of the PI in previous randomized controlled trials, and the power was 80% (G-power 3.0)^20^ Based on these results, a minimum sample size of 18 participants was determined. The total number of participants was calculated considering a dropout rate of approximately 20% of the study participants.

Participants were included in this study based on the following criteria: those who had a fixed orthodontic appliance for more than 1 month, had no systemic disease known to affect oral tissues, had no periodontal therapy, such as scaling, performed in the past 3 months, and had not used antibiotics or disinfectant mouthwashes for the past 1 month, selected participants with 20 or more teeth, and healthy participants without uncontrolled systemic disease. The exclusion criteria were as follows: a history of lack of oral hygiene maintenance, Loe and Silness’s GI above 2 points, presence of five or more untreated decayed teeth, a history of smoking, a history of medication with known adverse effects to gingival health, patients with uncontrolled systemic disease, refusal to provide informed consent and those deemed inappropriate as participants by the principal investigator.

**Figure 1:**
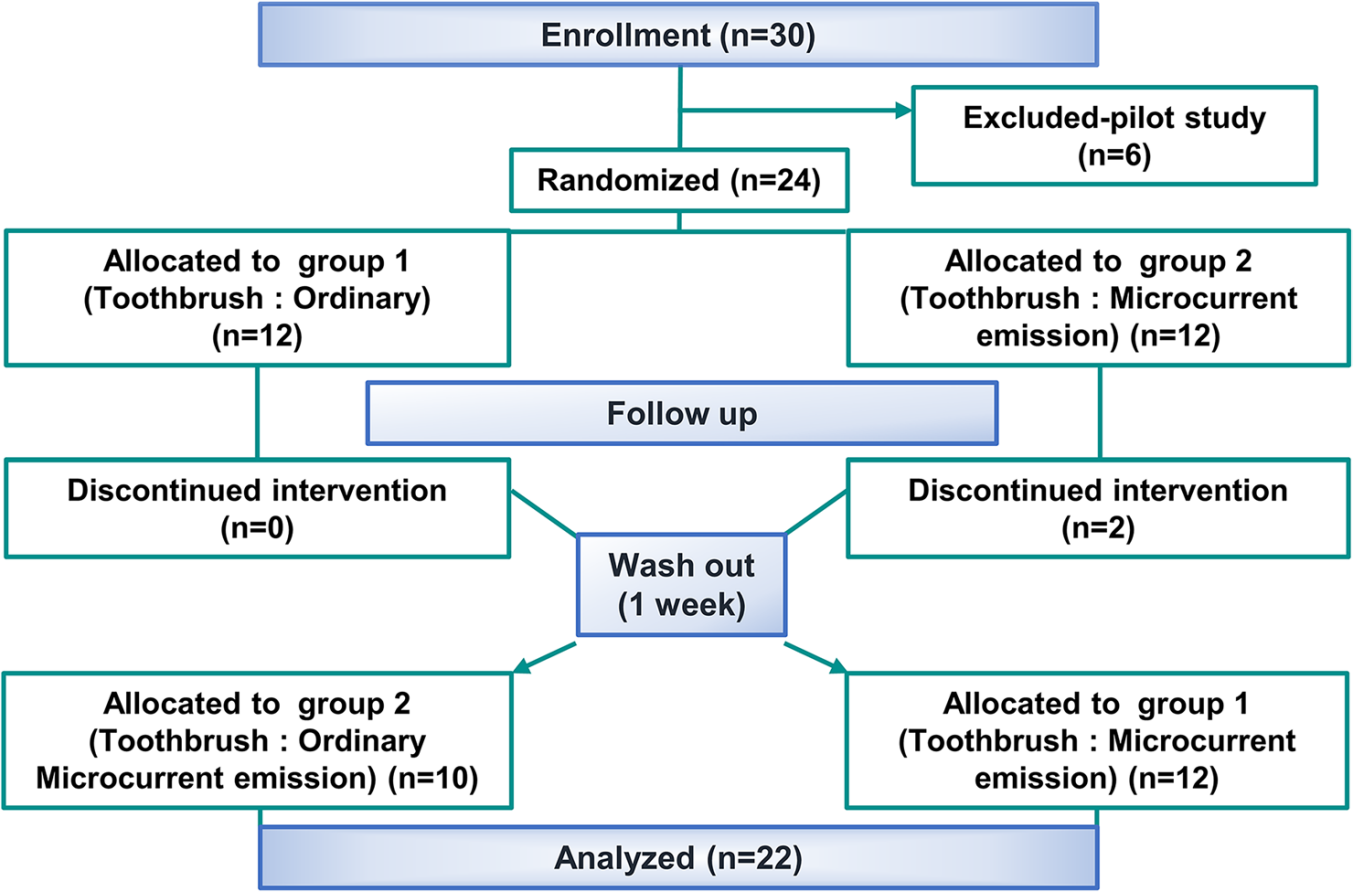
Crossover study design adapted for the evaluation of microcurrent-emitting toothbrush.

### Oral examination

Loe and Silness’s GI evaluation method was used to check whether the GI evaluation and participant selection criteria were met^20^

**Table 1:** Progress checks and procedures.

	Screening (T1-30)	Visit 1(T1)	Visit 2	Visit 3
Oral examination	√			
Specific instructions	√	√	√	
Tooth brushing instructions		√	√	
Dental plaque collection		√	√	√
Plaque index evaluation		√	√	√
Dental caries activity evaluation		√	√	√
Satisfaction assessment				√

**Table 2:** Loe and Silness’s (1963) gingival index criteria.

Score	Division	
0	Normal gingiva	Natural coral pink gingiva with no sign of inflammation
1	Mild inflammation	Slight change in color, slight edema. No bleeding on probing
2	Moderate inflammation	Redness, edema, and glazing. Bleeding upon probing
3	Severe inflammation	Severe inflammation: severe erythema and swelling tendency to spontaneous bleeding; possible ulceration

### Tooth brushing and other specific instructions

Participants were requested to continue brushing their teeth with the same toothpaste and refrain from using oral hygiene products other than toothbrushes during the clinical study. The participants' brushing methods were identified before the start of the study. The operator trained the participants using the Bass method (cross section of the bristles facing the gingiva, maintained at a 45° angle and wiped with vibration) for toothbrush introduction. Brushing was performed three times a day (morning, noon, and evening), and the brushing time was 2 min. Participants were also instructed to refrain from oral hygiene activities at 11 pm the day before the next visit and to avoid the consumption of water 1 h before the examination.

### PI evaluation

PI was evaluated using the Attin’s method where the disclosing agent is used to stain the plaque^1,21, 22^

**Table 3:** The Attin's plaque index evaluation method.

Score	
0	No visible plaque
1	Plaque buildup on the side of the bracket
2	Plaque accumulation in a single isolated form on the side of the bracket and on the cervical region
3	Plaque accumulation on the side of the bracket and one-third of the gingival surface

**Figure 2:**
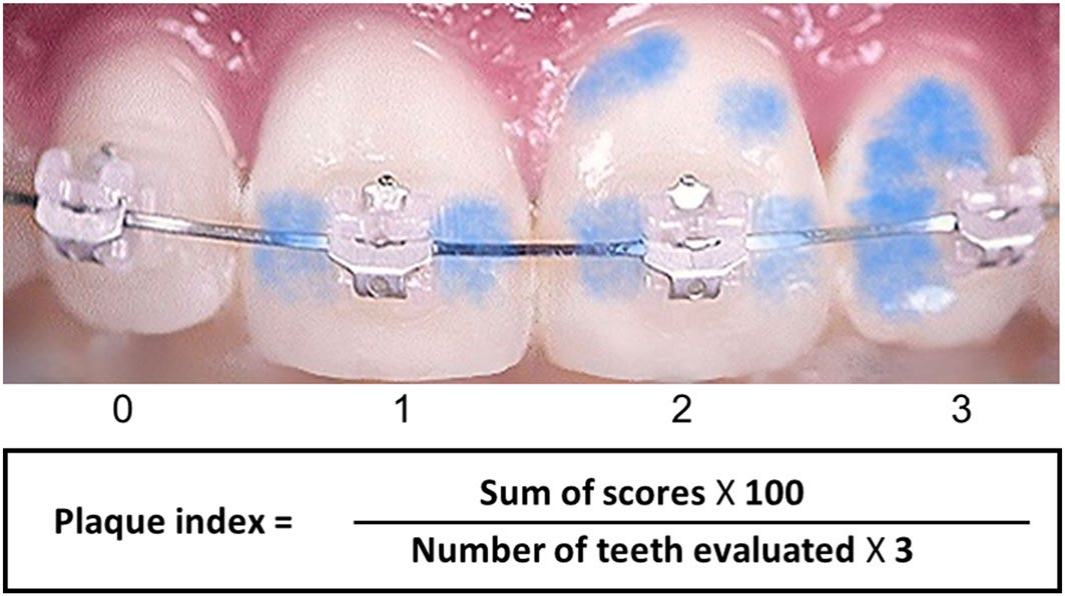
The classification of plaque index using the Attin's plaque index evaluation method.

### Caries activity test

A caries activity diagnostic kit (Cariview, Aiobio, Seoul, Korea) was used for caries risk assessment via colorimetric evaluation, which included quantifying and measuring the acidity of organic acids secreted by microorganisms within the plaque^21,22^. Plaques were collected 26 and 27 times with a cotton swab, and cotton was applied to tubes filled with the culture medium. The cells were incubated in a Hangil incubator (Hangil Science, Bucheon, Korea) at 37 °C for 48 h. After incubation, the cotton swab was removed, and 10 drops of the indicator were applied to the sample tube. The photograph was taken in an ample tube with an optical analyzer (Aiobio, Seoul Korea), and the image was transferred to the company on a website for calorimetric evaluation. The final caries activity was also calculated.

### Assessment of patient satisfaction

Satisfaction questionnaires regarding “freshness in mouth” and “cleansing degree” experienced by the participants were requested and compared after the use of both toothbrushes. Responses were graded on a five-point subjective scale ranging from excellent (most positive response) to worst (most negative response).

### Statistical analysis

The collected data were analyzed using IBM SPSS Statistics 5.0 (IBM Co., Armonk, NY, USA), and the statistical significance level was set at p < 0.05. Intraclass correlation coefficients were used to calculate the level of confidence among operators in assessing PI. The Shapiro–Wilk test confirmed that the data followed a normal distribution and were analyzed using parametric methods. Independent t-tests and chi-squared tests were used to compare the general characteristics of the two groups of study participants. An independent t-test was performed to verify the equivalence of the crossover, timing, and treatment effects between the two groups. The pre- and post-toothbrush use comparisons were performed using a paired t-test, and the difference between the two groups was compared using an independent t-test.

### Ethics declarations

This study was approved by the Institutional Bioethics Review Board of Yonsei University, Severance Hospital (IRB No. 2–2020-0089) and complied with the most recent Helsinki Declaration revised in 2013 regarding the planning and implementation of the study protocol.

## Results

### Characteristics of participants

**Table 4:** Demographic characteristics of participants.

Variable	Total (N = 22)	Group 1 (N = 12)	Group 2 (N = 10)	p-value
Age	23.6 ± 4.6	23.6 ± 4.2	23.4 ± 5.3	0.973
Male	11(50.0)	5 (41.7)	6 (60.0)	0.670
Female	11(50.0)	7 (58.3)	4 (40.0)	
Average number of brushings per day				
< 3	7 (31.8)	3 (25.0)	4 (40.0)	0.652
≥ 3	15 (68.2)	9 (75.0)	6 (60.0)	

### Intraclass correlation coefficients

The reliability of the evaluation methods for MCTs and conventional toothbrushes was measured. The intraclass correlation coefficient of the PI for six participants had a degree of agreement of 95.9% (p < 0.001).

### Treatment effect on PI within toothbrush groups

**Table 5:** The effects of the equivalence test on the design.

	Carry-over effect			Period effect			Treatment effect		
	Difference	confidence interval	p-value	Difference	confidence interval	p-value	Difference	confidence interval	p-value
Plaque index	1.2	− 6.8, 4.7	0.249	0.3	− 7.6, 10.4	0.745	2.8	2.9, 0.8	0.012*
Dental caries activity	−1.4	− 54.6, 10.4	0.172	0.8	− 14.1, 30.2	0.459	0.8	− 15.4, 31.7	0.451

### Treatment effect on dental caries activity within toothbrush groups.

The results of the carryover effect analysis showed no significant difference (p = 0.172). The timing effect analysis also confirmed that there was no significant difference (p = 0.459) for the choice of toothbrush at the beginning of the trial. In addition, it was confirmed that there was no significant difference (p = 0.451) in the treatment effect analysis. (Table 5).

### Analysis of changes in dental plaque index and dental caries activity between the toothbrush groups

**Figure 3:**
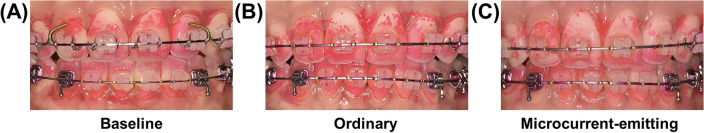
Representative photo of plaque index evaluation after clinical trial. (**A**) based on the beginning of the trial (score 64.58%) (**B**) after use of ordinary toothbrush (score 47.92%) (**C**) after use of microcurrent-emitting toothbrush (score 52.08%).

**Table 6:** Evaluation of change of dental plaque index and dental caries activity according to toothbrush type.

	Microcurrent-emitting (n = 22)			Ordinary(n = 22)			p-value^‡^
	Mean ± SD	Difference	p-value ^*†*^	Mean ± SD	Difference	p-value^*†*^	
Plaque index	67.4 ± 9.2	− 7.0	0.009	73.4 ± 7.9	− 1	0.595	0.058
Dental caries activity	59.8 ± 17.0	− 7.8	0.184	64.2 ± 5.0	− 3.4	0.571	0.589

### Patient satisfaction evaluation

**Table 7:** Distribution of satisfaction with microcurrent-emitting and ordinary toothbrushes.

Time	Questionnaire	Excellent	Good	Fair	Poor	Worst
Microcurrent-emitting	Freshness	0.5	2.75	1.5	0.25	0
	Cleansing degree	0.75	2	1.75	0.5	0
Ordinary	Freshness	0	2.5	2.5	0	0
	Cleansing degree	0	2	2.75	0.25	0

**Figure 4:**
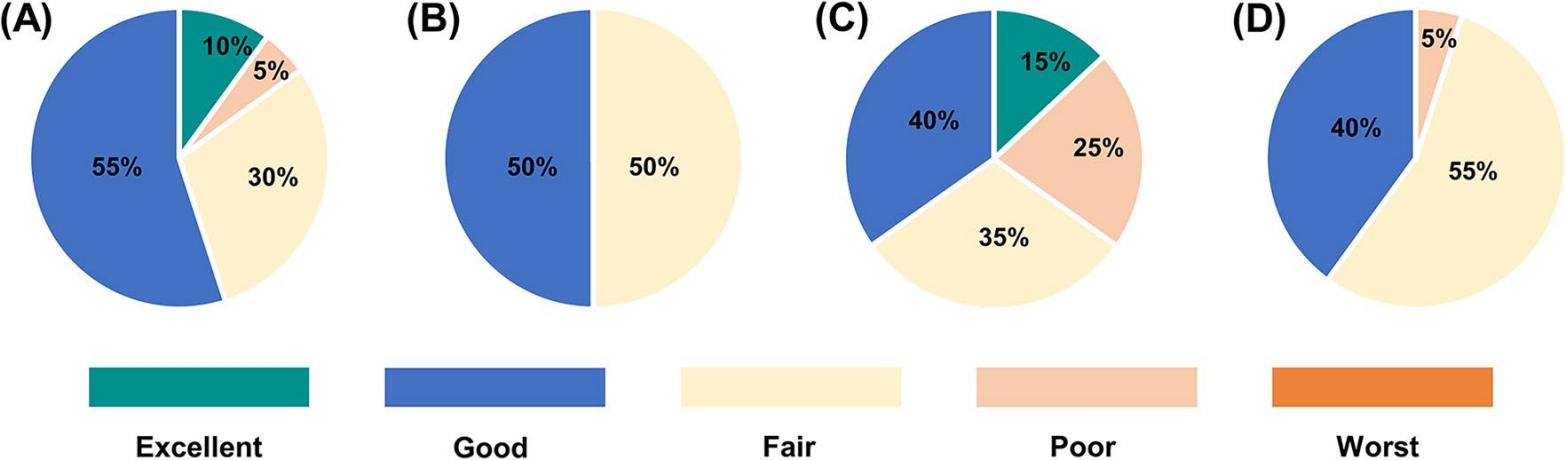
Comparison of satisfaction with use of microcurrent-emitting and ordinary toothbrushes. Results of freshness rated by the **(A)** microcurrent-emitting toothbrush group and **(B)** ordinary toothbrush group. Results of cleansing degree rated by the **(C)** microcurrent-emitting toothbrush group and **(D)** the ordinary toothbrush group.

## Discussion

In general, the treatment period for orthodontic patients is greater than 2 years. Considering the duration of treatment and number of patients receiving treatment, oral hygiene management for continuous plaque removal is important. In a clinical study involving human subjects, we utilized a crossover design study to compensate for the Hawthorne effect, which accounts for temporary changes in efficiency. This crossover study compared ordinary toothbrushes and MCTs for plaque control in orthodontic patients receiving fixed orthodontic treatments. In a crossover design, each participant used a toothbrush with a washout period (to eliminate residual effects) between the study periods. In total, 12 participants were included for Group 1 and 10 for Group 2, excluding two dropouts. As it was a crossover study, each group was evaluated with twice the sample size. This methodology has been widely used in toothbrush studies in orthodontic patients^20,23–27^. Therefore, in the present study, a crossover design was adopted to validate the in vitro effects of MCTs^16^.

This clinical study targeted adults in their twenties based on the assumption that orthodontic treatments generally take place between 15–24 years old, and thus did not investigate the effects across all age groups. In a toothbrush study targeting adults aged 18–50 years, there were no significant differences in PI evaluation results among the different age groups (ANOVA test, P value = 0.435)^28^. Oral hygiene practices decline in adults aged > 55 years old due to cognitive decline, decreased hand grip strength, muscle strength, and comorbid systemic conditions^29^. In children, auditory and visual task functioning improve with increasing age. A study evaluating PI at in children aged 8 and 9 years old reported that regardless of the method of oral health education, older children showed significant improvements in PI^30^. To minimize bias, this study targeted capable adults for oral hygiene assessment instead of younger pediatric patients, considering that functional oral hygiene capacity could act as a confounding variable in pediatric patients. Additionally, efforts were made to control research variables as much as possible to account for the Hawthorne effect. Future studies should target diverse age groups, including adolescents, to enhance the validity and accuracy of the efficacy of microcurrents in reducing PI.

According to a recent study on 40 individuals comparing MCTs and ordinary toothbrushes, the PI and GI were significantly reduced in the experimental group compared to the control group. The microcurrent that enhances adenosine triphosphate production reduces gingival inflammation in gingival tissue and tissue regeneration and induces growth factor expression to reduce gingivitis. The mechanism of gingivitis reduction suggests that there may be a synergistic effect between the reduction in biofilms and the microcurrent’s anti-inflammatory effect. The MCTs showed a significantly decreased biofilm in the proximal area compared to the ordinary toothbrush and was observed to have a beneficial effect on the interdental area where the bristles of the toothbrush do not reach^31^.

The mechanism of the BE effect is based, in part, on an external electric field that alters bacterial cell membranes containing a variety of molecules, including cellular proteins, polysaccharides, nucleic acids, and lipids^31–34^. Whereas other electric current-based methods focus on a single mechanism (direct current or alternating current), the corresponding microcurrent combines the two signals to maximize the effect^35–41^. When direct current and alternating current are simultaneously applied to the biofilm, its metabolic stress rapidly increases, causing BE effects according to the electrostatic force, medium electrolysis, enzyme inactivation, nonuniform electrolyte distribution, and changes in the electrochemical environment, resulting in biofilm reduction.

This study targeted the area around the orthodontic appliances for evaluating plaque reduction efficacy. To minimize variability related to different bracket types, ceramic brackets were used. Gomes et al.^42^^43^ reported that there was no significant difference in plaque index (PI), gingival index (GI), bleeding on probing (BOP), or microbiological analysis results between conventional and self-ligating brackets. Pandis et al.^44^^45^ and Nalçacı et al.^46^ reported that conventional brackets had a higher rate of plaque accumulation compared to self-ligating brackets, and Issa. et al.^47^ reported significant differences in plaque index among conventional, ceramic, and clear brackets.

Our results somewhat differ from previous studies. Patients undergoing orthodontic treatment with self-ligating brackets may have difficulty maintaining oral hygiene due to the structure of the appliance, leading to increased plaque accumulation and gingivitis risk during the two-year treatment period. According to a study performed by Lombardo et al.^48^, orthodontic patients who received self-ligating brackets experience more difficulties in maintaining their oral hygiene when compared to those with conventional brackets. Artyn et al.^49^ and Sinclair et al.^50^ also reported noticeable plaque accumulation in patients with self-ligating brackets. Since the oral cavity is an electrically conductive medium, it is possible that different bracket types may influence the transmission of electromagnetic waves through the dielectric constant (εr = 80), although this is currently unproven^14^. Future studies should aim to investigate the effect of bracket types and materials on plaque reduction through bioelectrical effects.

The analysis of satisfaction surveys confirmed the subjective effect of MCTs in improving oral health. The MCT group had a higher satisfaction level regarding cleanliness, but the difference was not significant. Regarding freshness, the MCT group had a 65% score in the excellent and good categories, compared to 45% for the ordinary toothbrushes. In terms of the cleansing capability, the MCT group had a 50% score in the excellent and good categories, compared to 40% for ordinary toothbrushes.

A comparative analysis of the PI data of the MCT and the ordinary toothbrush used for 4 weeks based on the baseline (before brushing) showed that the tooth surface bacteria decreased with use of MCT, by 7.1 points (a 9.43% decrease), whereas the ordinary toothbrush showed a limited change of 1.1 points (a 1.42% decrease). Therefore, the comparative analysis indicated an improved efficacy of MCTs in PI reduction.

Caries activity test analysis showed a 11.59% decrease (by 7.8 points) in dental caries activity (from 67.6 to 59.8 points) after use of MCTs. In contrast, a 5% decrease (67.6 to 64.20 points; 3.4-point decrease) was observed with use of an ordinary toothbrush. Statistical comparisons with the base group were not significant in either group. In addition, our results did not show any significant difference between the two toothbrush groups.

A decrease in PI was observed when MCTs were used. Considering that it is difficult for orthodontic patients to remove plaque due to fixed attachments, it can be seen that using MCTs can be a way to effectively maintain oral care. However, there was no significant decrease in the caries activity. However, this was a short-term study using a randomized crossover method, and long-term clinical trials need to be performed to support the results of this study with long-term effects.

In this study, there was no significant difference in caries activity when compared to the control group which used regular toothbrushes. This suggests that the BE-emitting toothbrush might have reduced the biofilm volume in the early stages by affecting the attachment of EPS to the biofilm structure, leading to decreased biofilm formation. This is consistent with the 9.43% decrease in plaque index (PI) observed in our study when using MCTs. In a long-term evaluation of caries activity among orthodontic patients, the increase in caries activity observed in patients undergoing orthodontic treatment is attributed to the difficulty in performing oral hygiene practices and long-term plaque accumulation during the orthodontic treatment period^51^. Our study had some limitations, including a short duration (four weeks). Since oral hygiene education was provided to all groups before the start of the study, this short duration may have only minimally impacted caries activity. However, further research on the long-term effects of various interventions are needed, to ensure the validity and accuracy of studies which compare caries activity.

This study showed that MCTs were more effective in reducing the dental PI than ordinary toothbrushes in orthodontic patients. However, there was no significant difference in dental caries activity between the two groups. The patient satisfaction assessment revealed a higher level of freshness with MCTs than with ordinary toothbrushes. These findings suggest that MCTs are a beneficial addition to oral hygiene practices for orthodontic patients as the use of MCTs may lead to improved plaque control and a reduced risk of periodontal disease. Additionally, this study adds to the body of evidence on the effectiveness of microcurrent technology in oral health care, which suggests a synergistic effect of the reduction of biofilms and the microcurrent anti-inflammatory.

## Data Availability

Data supporting the findings of this study are available from the corresponding author upon request.
