# Author Correction: Evaluation of dental plaque reduction using microcurrent-emitting toothbrushes in orthodontic patients: a randomized, double-blind, crossover clinical trial

**DOI:** 10.1038/s41598-024-68625-y

**Published:** 2024-08-08

**Authors:** Ji-Hoi Kim, Jae-Hun Yu, Utkarsh Mangal, Jing Liu, Hyo-Jung Jung, Jung-Yul Cha

**Affiliations:** 1 Department of Orthodontics, Institute of Craniofacial Deformity, Yonsei University College of Dentistry, Seoul, Korea; 2 BK21 FOUR Project, Yonsei University College of Dentistry, Seoul, Korea; 3 Department of Orofacial Pain and Oral Medicine, Dental Hospital, Yonsei University College of Dentistry, Seoul, Korea; 4 Department of Orthodontics, Institute of Craniofacial Deformity, College of Dentistry, Institute for Innovation in Digital Healthcare, Yonsei University, Seoul, Korea

Correction to: *Scientific Reports* 10.1038/s41598-024-60753-9, published online 27 May 2024

The original version of this Article contained an error in Figure 2, where a parenthesis was erroneously included in the equation. The original Figure 2 and accompanying legend appear below.

**Figure 2:**
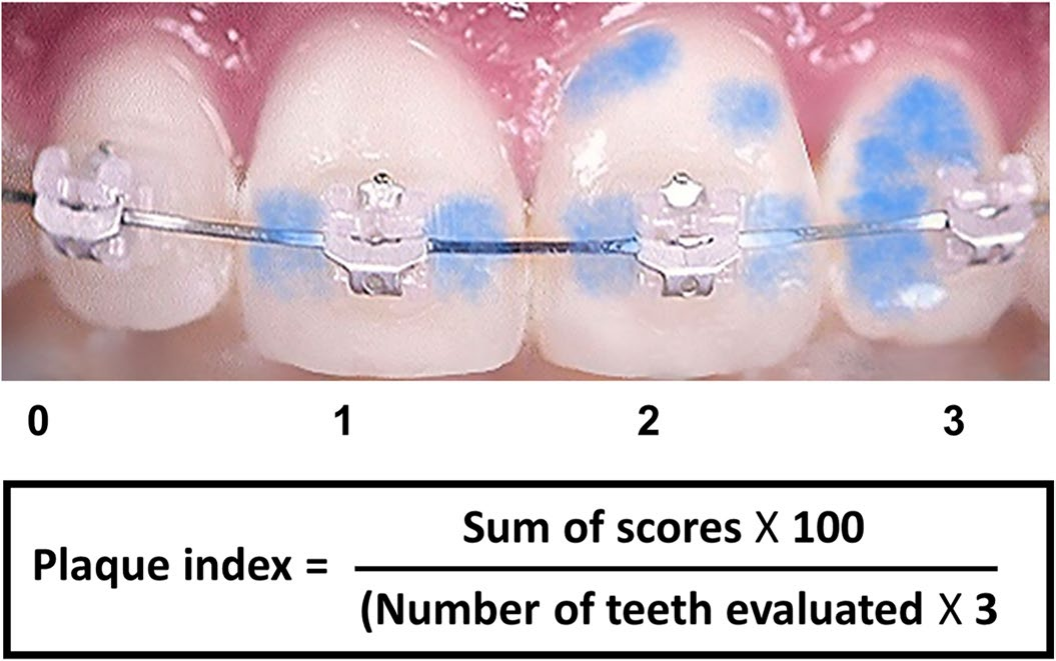
The classification of plaque index using the Attin's plaque index evaluation method.

The original Article has been corrected.

